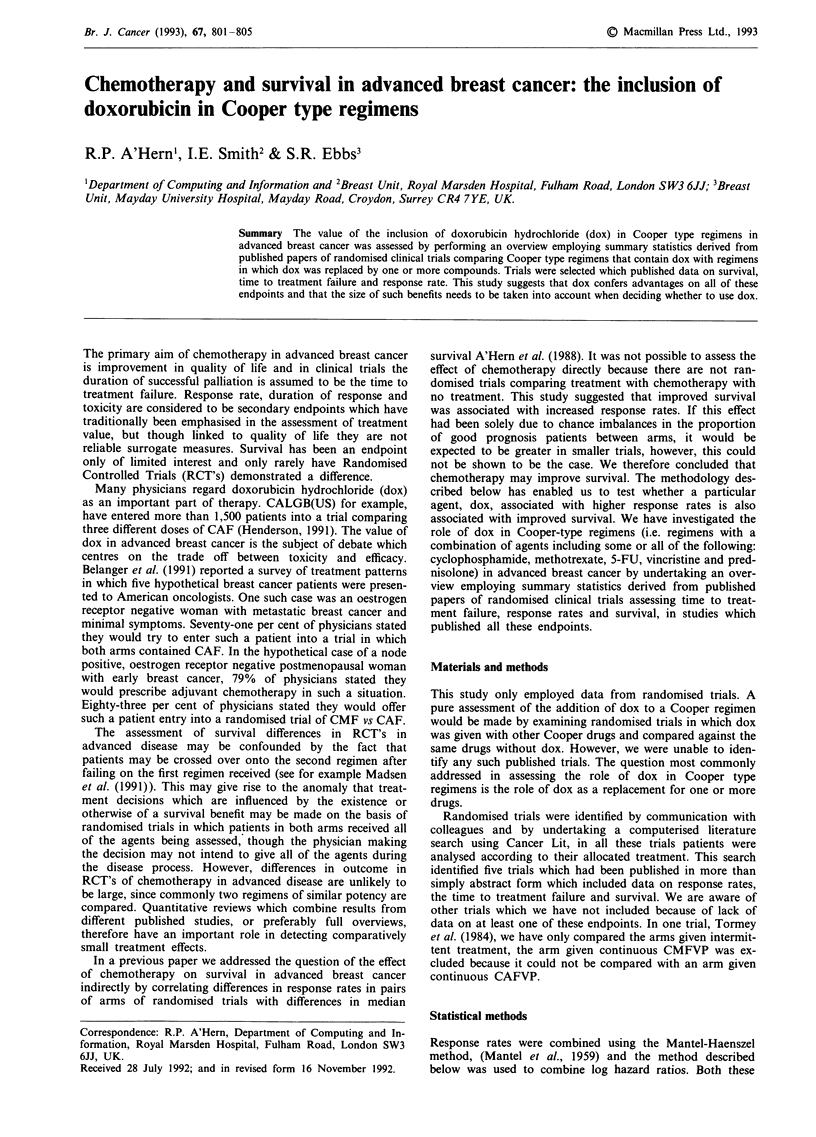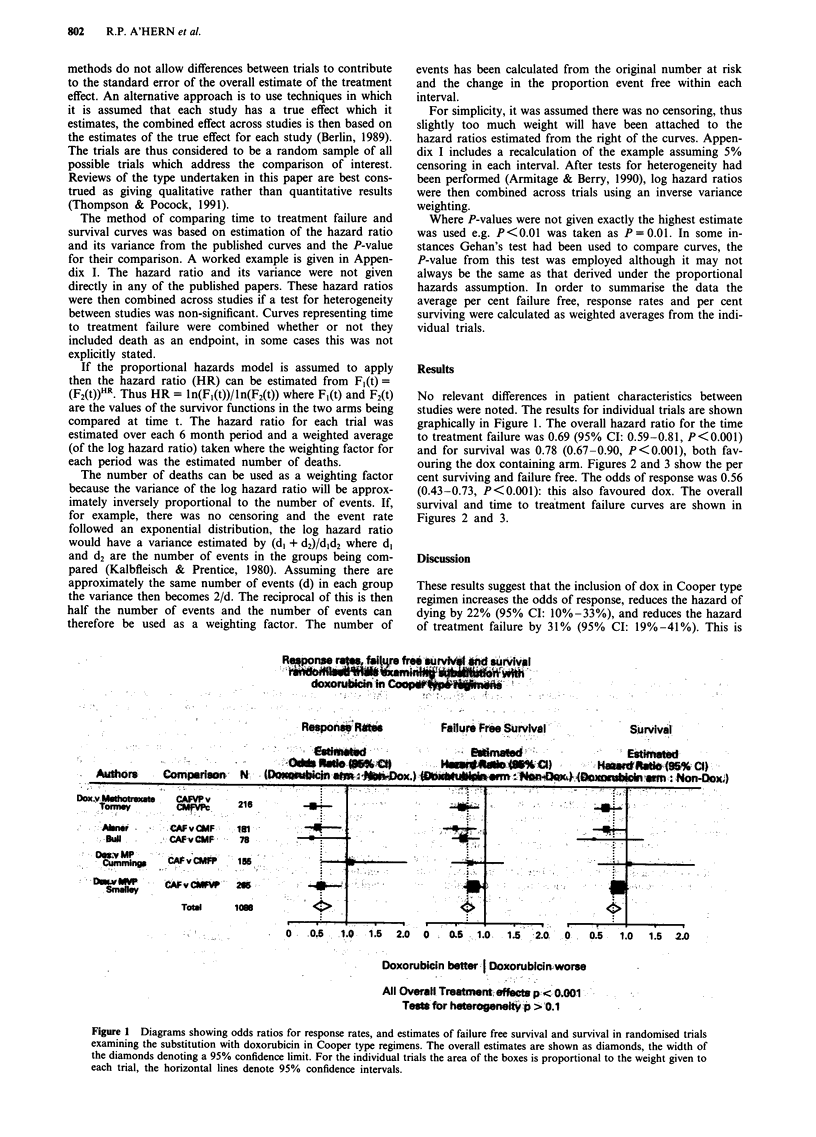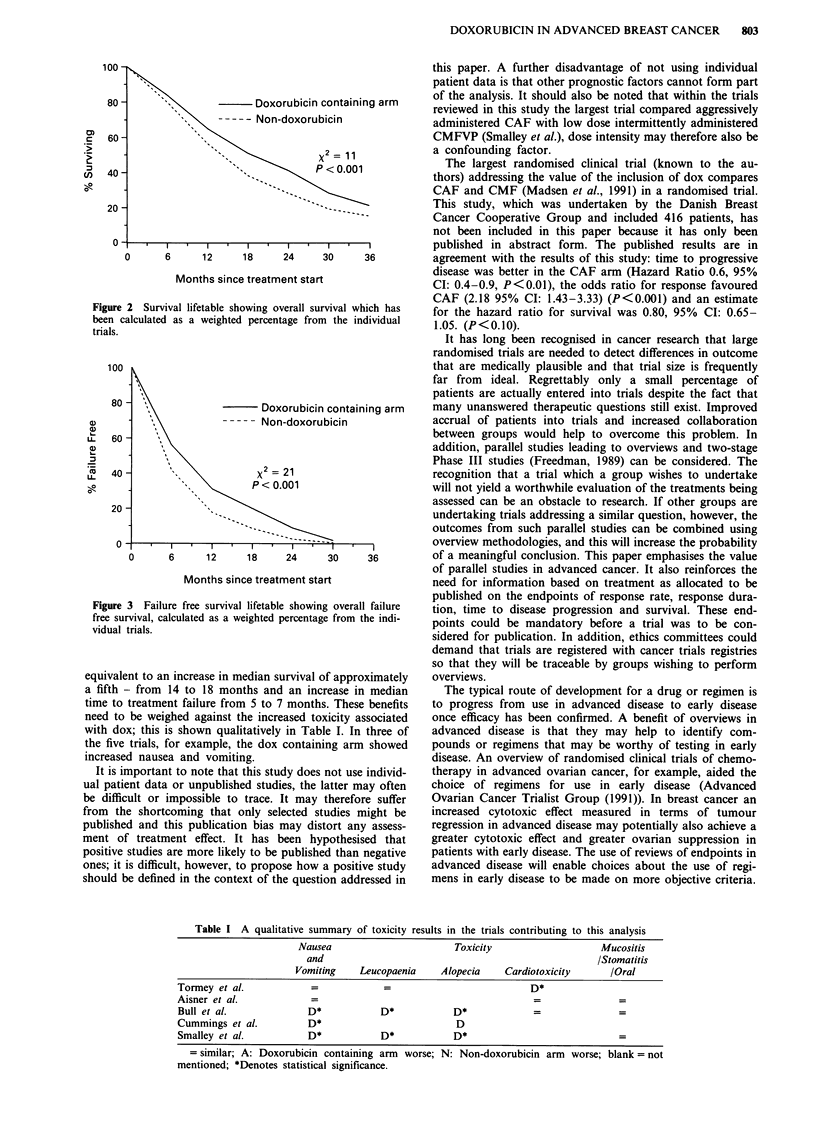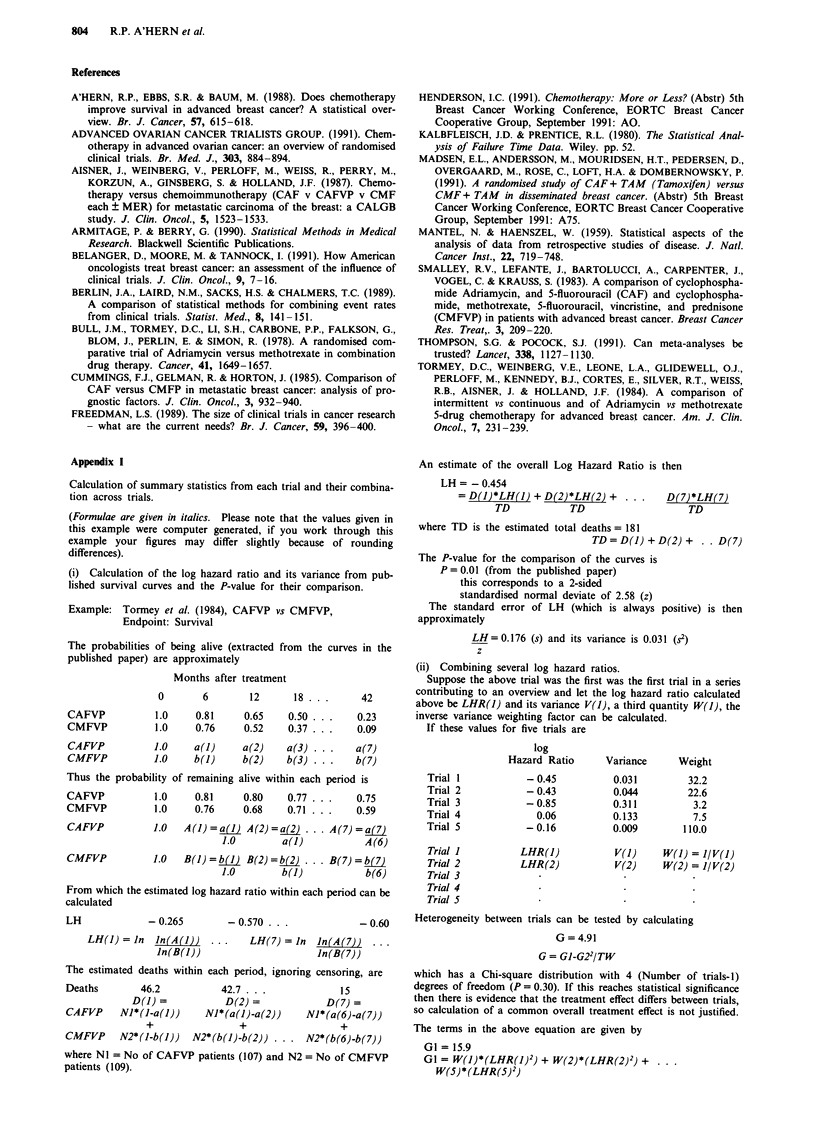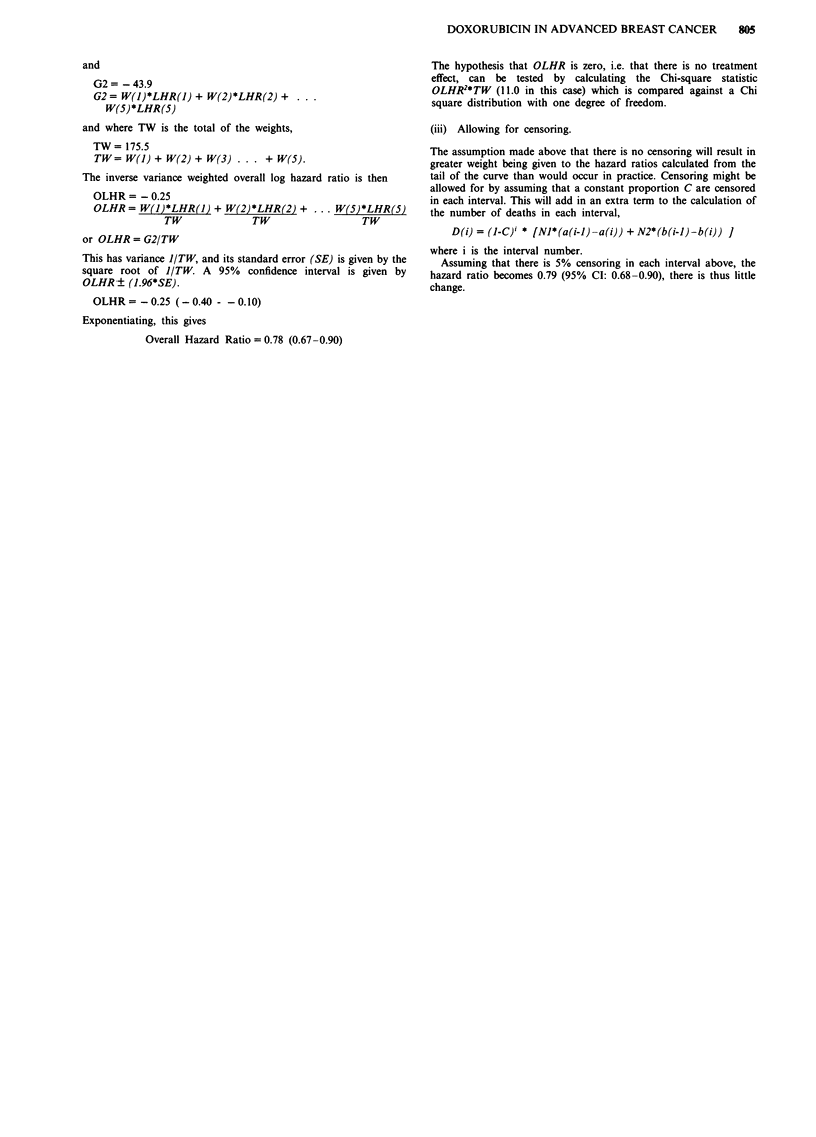# Chemotherapy and survival in advanced breast cancer: the inclusion of doxorubicin in Cooper type regimens.

**DOI:** 10.1038/bjc.1993.146

**Published:** 1993-04

**Authors:** R. P. A'Hern, I. E. Smith, S. R. Ebbs

**Affiliations:** Department of Computing and Information, Royal Marsden Hospital, London, UK.

## Abstract

The value of the inclusion of doxorubicin hydrochloride (dox) in Cooper type regimens in advanced breast cancer was assessed by performing an overview employing summary statistics derived from published papers of randomised clinical trials comparing Cooper type regimens that contain dox with regimens in which dox was replaced by one or more compounds. Trials were selected which published data on survival, time to treatment failure and response rate. This study suggests that dox confers advantages on all of these endpoints and that the size of such benefits needs to be taken into account when deciding whether to use dox.


Br. J. Cancer (1993), 67, 801-805 © Macmillan Press Ltd., 1993

### Chemotherapy and survival in advanced breast cancer: the inclusion of doxorubicin in Cooper type regimens

R.P. A'Hern[1], I.E. Smith[2] & S.R. Ebbs[3]

[1]*Department of Computing and Information and* [2]*Breast Unit, Royal Marsden Hospital, Fulham Road, London SW3 6JJ;* [3]*Breast Unit, Mayday University Hospital, Mayday Road, Croydon, Surrey CR4 7YE, UK.*

**Summary** The value of the inclusion of doxorubicin hydrochloride (dox) in Cooper type regimens in advanced breast cancer was assessed by performing an overview employing summary statistics derived from published papers of randomised clinical trials comparing Cooper type regimens that contain dox with regimens in which dox was replaced by one or more compounds. Trials were selected which published data on survival, time to treatment failure and response rate. This study suggests that dox confers advantages on all of these endpoints and that the size of such benefits needs to be taken into account when deciding whether to use dox.

The primary aim of chemotherapy in advanced breast cancer is improvement in quality of life and in clinical trials the duration of successful palliation is assumed to be the time to treatment failure. Response rate, duration of response and toxicity are considered to be secondary endpoints which have traditionally been emphasised in the assessment of treatment value, but though linked to quality of life they are not reliable surrogate measures. Survival has been an endpoint only of limited interest and only rarely have Randomised Controlled Trials (RCT's) demonstrated a difference.

Many physicians regard doxorubicin hydrochloride (dox) as an important part of therapy. CALGB(US) for example, have entered more than 1,500 patients into a trial comparing three different doses of CAF (Henderson, 1991). The value of dox in advanced breast cancer is the subject of debate which centres on the trade off between toxicity and efficacy. Belanger *et al.* (1991) reported a survey of treatment patterns in which five hypothetical breast cancer patients were presented to American oncologists. One such case was an oestrogen receptor negative woman with metastatic breast cancer and minimal symptoms. Seventy-one per cent of physicians stated they would try to enter such a patient into a trial in which both arms contained CAF. In the hypothetical case of a node positive, oestrogen receptor negative postmenopausal woman with early breast cancer, 79% of physicians stated they would prescribe adjuvant chemotherapy in such a situation. Eighty-three per cent of physicians stated they would offer such a patient entry into a randomised trial of CMF *vs* CAF.

The assessment of survival differences in RCT's in advanced disease may be confounded by the fact that patients may be crossed over onto the second regimen after failing on the first regimen received (see for example Madsen *et al.* (1991)). This may give rise to the anomaly that treatment decisions which are influenced by the existence or otherwise of a survival benefit may be made on the basis of randomised trials in which patients in both arms received all of the agents being assessed, though the physician making the decision may not intend to give all of the agents during the disease process. However, differences in outcome in RCT's of chemotherapy in advanced disease are unlikely to be large, since commonly two regimens of similar potency are compared. Quantitative reviews which combine results from different published studies, or preferably full overviews, therefore have an important role in detecting comparatively small treatment effects.

In a previous paper we addressed the question of the effect of chemotherapy on survival in advanced breast cancer indirectly by correlating differences in response rates in pairs of arms of randomised trials with differences in median survival A'Hern *et al.* (1988). It was not possible to assess the effect of chemotherapy directly because there are not randomised trials comparing treatment with chemotherapy with no treatment. This study suggested that improved survival was associated with increased response rates. If this effect had been solely due to chance imbalances in the proportion of good prognosis patients between arms, it would be expected to be greater in smaller trials, however, this could not be shown to be the case. We therefore concluded that chemotherapy may improve survival. The methodology described below has enabled us to test whether a particular agent, dox, associated with higher response rates is also associated with improved survival. We have investigated the role of dox in Cooper-type regimens (i.e. regimens with a combination of agents including some or all of the following: cyclophosphamide, methotrexate, 5-FU, vincristine and prednisolone) in advanced breast cancer by undertaking an overview employing summary statistics derived from published papers of randomised clinical trials assessing time to treatment failure, response rates and survival, in studies which published all these endpoints.

### Materials and methods

This study only employed data from randomised trials. A pure assessment of the addition of dox to a Cooper regimen would be made by examining randomised trials in which dox was given with other Cooper drugs and compared against the same drugs without dox. However, we were unable to identify any such published trials. The question most commonly addressed in assessing the role of dox in Cooper type regimens is the role of dox as a replacement for one or more drugs.

Randomised trials were identified by communication with colleagues and by undertaking a computerised literature search using Cancer Lit, in all these trials patients were analysed according to their allocated treatment. This search identified five trials which had been published in more than simply abstract form which included data on response rates, the time to treatment failure and survival. We are aware of other trials which we have not included because of lack of data on at least one of these endpoints. In one trial, Tormey *et al.* (1984), we have only compared the arms given intermittent treatment, the arm given continuous CMFVP was excluded because it could not be compared with an arm given continuous CAFVP.

### Statistical methods

Response rates were combined using the Mantel-Haenszel method, (Mantel *et al.*, 1959) and the method described below was used to combine log hazard ratios. Both these

Correspondence: R.P. A'Hern, Department of Computing and Information, Royal Marsden Hospital, Fulham Road, London SW3 6JJ, UK.
Received 28 July 1992; and in revised form 16 November 1992.

methods do not allow differences between trials to contribute to the standard error of the overall estimate of the treatment effect. An alternative approach is to use techniques in which it is assumed that each study has a true effect which it estimates, the combined effect across studies is then based on the estimates of the true effect for each study (Berlin, 1989). The trials are thus considered to be a random sample of all possible trials which address the comparison of interest. Reviews of the type undertaken in this paper are best construed as giving qualitative rather than quantitative results (Thompson & Pocock, 1991).

The method of comparing time to treatment failure and survival curves was based on estimation of the hazard ratio and its variance from the published curves and the *P*-value for their comparison. A worked example is given in Appendix I. The hazard ratio and its variance were not given directly in any of the published papers. These hazard ratios were then combined across studies if a test for heterogeneity between studies was non-significant. Curves representing time to treatment failure were combined whether or not they included death as an endpoint, in some cases this was not explicitly stated.

If the proportional hazards model is assumed to apply then the hazard ratio (HR) can be estimated from $F_1(t) = (F_2(t))^{HR}$. Thus $HR = \ln(F_1(t))/\ln(F_2(t))$ where $F_1(t)$ and $F_2(t)$ are the values of the survivor functions in the two arms being compared at time t. The hazard ratio for each trial was estimated over each 6 month period and a weighted average (of the log hazard ratio) taken where the weighting factor for each period was the estimated number of deaths.

The number of deaths can be used as a weighting factor because the variance of the log hazard ratio will be approximately inversely proportional to the number of events. If, for example, there was no censoring and the event rate followed an exponential distribution, the log hazard ratio would have a variance estimated by $(d_1 + d_2)/d_1d_2$ where $d_1$ and $d_2$ are the number of events in the groups being compared (Kalbfleisch & Prentice, 1980). Assuming there are approximately the same number of events (d) in each group the variance then becomes 2/d. The reciprocal of this is then half the number of events and the number of events can therefore be used as a weighting factor. The number of events has been calculated from the original number at risk and the change in the proportion event free within each interval.

For simplicity, it was assumed there was no censoring, thus slightly too much weight will have been attached to the hazard ratios estimated from the right of the curves. Appendix I includes a recalculation of the example assuming 5% censoring in each interval. After tests for heterogeneity had been performed (Armitage & Berry, 1990), log hazard ratios were then combined across trials using an inverse variance weighting.

Where *P*-values were not given exactly the highest estimate was used e.g. $P < 0.01$ was taken as $P = 0.01$. In some instances Gehan's test had been used to compare curves, the *P*-value from this test was employed although it may not always be the same as that derived under the proportional hazards assumption. In order to summarise the data the average per cent failure free, response rates and per cent surviving were calculated as weighted averages from the individual trials.

### Results

No relevant differences in patient characteristics between studies were noted. The results for individual trials are shown graphically in Figure 1. The overall hazard ratio for the time to treatment failure was 0.69 (95% CI: 0.59–0.81, $P < 0.001$) and for survival was 0.78 (0.67–0.90, $P < 0.001$), both favouring the dox containing arm. Figures 2 and 3 show the per cent surviving and failure free. The odds of response was 0.56 (0.43–0.73, $P < 0.001$): this also favoured dox. The overall survival and time to treatment failure curves are shown in Figures 2 and 3.

### Discussion

These results suggest that the inclusion of dox in Cooper type regimen increases the odds of response, reduces the hazard of dying by 22% (95% CI: 10%–33%), and reduces the hazard of treatment failure by 31% (95% CI: 19%–41%). This is

**Figure 1** Diagrams showing odds ratios for response rates, and estimates of failure free survival and survival in randomised trials examining the substitution with doxorubicin in Cooper type regimens. The overall estimates are shown as diamonds, the width of the diamonds denoting a 95% confidence limit. For the individual trials the area of the boxes is proportional to the weight given to each trial, the horizontal lines denote 95% confidence intervals.

**Figure 2** Survival lifetable showing overall survival which has been calculated as a weighted percentage from the individual trials.

**Figure 3** Failure free survival lifetable showing overall failure free survival, calculated as a weighted percentage from the individual trials.

equivalent to an increase in median survival of approximately a fifth - from 14 to 18 months and an increase in median time to treatment failure from 5 to 7 months. These benefits need to be weighed against the increased toxicity associated with dox; this is shown qualitatively in Table I. In three of the five trials, for example, the dox containing arm showed increased nausea and vomiting.

It is important to note that this study does not use individual patient data or unpublished studies, the latter may often be difficult or impossible to trace. It may therefore suffer from the shortcoming that only selected studies might be published and this publication bias may distort any assessment of treatment effect. It has been hypothesised that positive studies are more likely to be published than negative ones; it is difficult, however, to propose how a positive study should be defined in the context of the question addressed in this paper. A further disadvantage of not using individual patient data is that other prognostic factors cannot form part of the analysis. It should also be noted that within the trials reviewed in this study the largest trial compared aggressively administered CAF with low dose intermittently administered CMFVP (Smalley *et al.*), dose intensity may therefore also be a confounding factor.

The largest randomised clinical trial (known to the authors) addressing the value of the inclusion of dox compares CAF and CMF (Madsen *et al.*, 1991) in a randomised trial. This study, which was undertaken by the Danish Breast Cancer Cooperative Group and included 416 patients, has not been included in this paper because it has only been published in abstract form. The published results are in agreement with the results of this study: time to progressive disease was better in the CAF arm (Hazard Ratio 0.6, 95% CI: 0.4–0.9, $P<0.01$), the odds ratio for response favoured CAF (2.18 95% CI: 1.43–3.33) ($P<0.001$) and an estimate for the hazard ratio for survival was 0.80, 95% CI: 0.65–1.05. ($P<0.10$).

It has long been recognised in cancer research that large randomised trials are needed to detect differences in outcome that are medically plausible and that trial size is frequently far from ideal. Regrettably only a small percentage of patients are actually entered into trials despite the fact that many unanswered therapeutic questions still exist. Improved accrual of patients into trials and increased collaboration between groups would help to overcome this problem. In addition, parallel studies leading to overviews and two-stage Phase III studies (Freedman, 1989) can be considered. The recognition that a trial which a group wishes to undertake will not yield a worthwhile evaluation of the treatments being assessed can be an obstacle to research. If other groups are undertaking trials addressing a similar question, however, the outcomes from such parallel studies can be combined using overview methodologies, and this will increase the probability of a meaningful conclusion. This paper emphasises the value of parallel studies in advanced cancer. It also reinforces the need for information based on treatment as allocated to be published on the endpoints of response rate, response duration, time to disease progression and survival. These endpoints could be mandatory before a trial was to be considered for publication. In addition, ethics committees could demand that trials are registered with cancer trials registries so that they will be traceable by groups wishing to perform overviews.

The typical route of development for a drug or regimen is to progress from use in advanced disease to early disease once efficacy has been confirmed. A benefit of overviews in advanced disease is that they may help to identify compounds or regimens that may be worthy of testing in early disease. An overview of randomised clinical trials of chemotherapy in advanced ovarian cancer, for example, aided the choice of regimens for use in early disease (Advanced Ovarian Cancer Trialist Group (1991)). In breast cancer an increased cytotoxic effect measured in terms of tumour regression in advanced disease may potentially also achieve a greater cytotoxic effect and greater ovarian suppression in patients with early disease. The use of reviews of endpoints in advanced disease will enable choices about the use of regimens in early disease to be made on more objective criteria.

**Table I** A qualitative summary of toxicity results in the trials contributing to this analysis

| | *Nausea and Vomiting* | *Leucopaenia* | *Toxicity* *Alopecia* | *Cardiotoxicity* | *Mucositis /Stomatitis /Oral* |
|---|---|---|---|---|---|
| Tormey *et al.* | = | = | | D* | |
| Aisner *et al.* | = | | | = | = |
| Bull *et al.* | D* | D* | D* | = | = |
| Cummings *et al.* | D* | | D | | |
| Smalley *et al.* | D* | D* | D* | | = |

= similar; A: Doxorubicin containing arm worse; N: Non-doxorubicin arm worse; blank = not mentioned; *Denotes statistical significance.

### References

A'HERN, R.P., EBBS, S.R. & BAUM, M. (1988). Does chemotherapy improve survival in advanced breast cancer? A statistical overview. *Br. J. Cancer*, **57**, 615–618.

ADVANCED OVARIAN CANCER TRIALISTS GROUP. (1991). Chemotherapy in advanced ovarian cancer: an overview of randomised clinical trials. *Br. Med. J.*, **303**, 884–894.

AISNER, J., WEINBERG, V., PERLOFF, M., WEISS, R., PERRY, M., KORZUN, A., GINSBERG, S. & HOLLAND, J.F. (1987). Chemotherapy versus chemoimmunotherapy (CAF v CAFVP v CMF each ± MER) for metastatic carcinoma of the breast: a CALGB study. *J. Clin. Oncol.*, **5**, 1523–1533.

ARMITAGE, P. & BERRY, G. (1990). *Statistical Methods in Medical Research*. Blackwell Scientific Publications.

BELANGER, D., MOORE, M. & TANNOCK, I. (1991). How American oncologists treat breast cancer: an assessment of the influence of clinical trials. *J. Clin. Oncol.*, **9**, 7–16.

BERLIN, J.A., LAIRD, N.M., SACKS, H.S. & CHALMERS, T.C. (1989). A comparison of statistical methods for combining event rates from clinical trials. *Statist. Med.*, **8**, 141–151.

BULL, J.M., TORMEY, D.C., LI, S.H., CARBONE, P.P., FALKSON, G., BLOM, J., PERLIN, E. & SIMON, R. (1978). A randomised comparative trial of Adriamycin versus methotrexate in combination drug therapy. *Cancer*, **41**, 1649–1657.

CUMMINGS, F.J., GELMAN, R. & HORTON, J. (1985). Comparison of CAF versus CMFP in metastatic breast cancer: analysis of prognostic factors. *J. Clin. Oncol.*, **3**, 932–940.

FREEDMAN, L.S. (1989). The size of clinical trials in cancer research – what are the current needs? *Br. J. Cancer*, **59**, 396–400.

HENDERSON, I.C. (1991). *Chemotherapy: More or Less?* (Abstr) 5th Breast Cancer Working Conference, EORTC Breast Cancer Cooperative Group, September 1991: AO.

KALBFLEISCH, J.D. & PRENTICE, R.L. (1980). *The Statistical Analysis of Failure Time Data*. Wiley. pp. 52.

MADSEN, E.L., ANDERSSON, M., MOURIDSEN, H.T., PEDERSEN, D., OVERGAARD, M., ROSE, C., LOFT, H.A. & DOMBERNOWSKY, P. (1991). *A randomised study of CAF + TAM (Tamoxifen) versus CMF + TAM in disseminated breast cancer.* (Abstr) 5th Breast Cancer Working Conference, EORTC Breast Cancer Cooperative Group, September 1991: A75.

MANTEL, N. & HAENSZEL, W. (1959). Statistical aspects of the analysis of data from retrospective studies of disease. *J. Natl. Cancer Inst.*, **22**, 719–748.

SMALLEY, R.V., LEFANTE, J., BARTOLUCCI, A., CARPENTER, J., VOGEL, C. & KRAUSS, S. (1983). A comparison of cyclophosphamide Adriamycin, and 5-fluorouracil (CAF) and cyclophosphamide, methotrexate, 5-fluorouracil, vincristine, and prednisone (CMFVP) in patients with advanced breast cancer. *Breast Cancer Res. Treat*,. **3**, 209–220.

THOMPSON, S.G. & POCOCK, S.J. (1991). Can meta-analyses be trusted? *Lancet*, **338**, 1127–1130.

TORMEY, D.C., WEINBERG, V.E., LEONE, L.A., GLIDEWELL, O.J., PERLOFF, M., KENNEDY, B.J., CORTES, E., SILVER, R.T., WEISS, R.B., AISNER, J. & HOLLAND, J.F. (1984). A comparison of intermittent *vs* continuous and of Adriamycin *vs* methotrexate 5-drug chemotherapy for advanced breast cancer. *Am. J. Clin. Oncol.*, **7**, 231–239.

### Appendix I

Calculation of summary statistics from each trial and their combination across trials.

(*Formulae are given in italics.* Please note that the values given in this example were computer generated, if you work through this example your figures may differ slightly because of rounding differences).

(i) Calculation of the log hazard ratio and its variance from published survival curves and the *P*-value for their comparison.

Example: Tormey *et al.* (1984), CAFVP *vs* CMFVP,
Endpoint: Survival

The probabilities of being alive (extracted from the curves in the published paper) are approximately

| | Months after treatment | | | | |
|---|---|---|---|---|---|
| | 0 | 6 | 12 | 18 . . . | 42 |
| CAFVP | 1.0 | 0.81 | 0.65 | 0.50 . . . | 0.23 |
| CMFVP | 1.0 | 0.76 | 0.52 | 0.37 . . . | 0.09 |
| *CAFVP* | *1.0* | *a(1)* | *a(2)* | *a(3) . . .* | *a(7)* |
| *CMFVP* | *1.0* | *b(1)* | *b(2)* | *b(3) . . .* | *b(7)* |

Thus the probability of remaining alive within each period is

| | | | | | |
|---|---|---|---|---|---|
| CAFVP | 1.0 | 0.81 | 0.80 | 0.77 . . . | 0.75 |
| CMFVP | 1.0 | 0.76 | 0.68 | 0.71 . . . | 0.59 |

*CAFVP* $1.0 \quad A(1) = \frac{a(1)}{1.0} \quad A(2) = \frac{a(2)}{a(1)} \; . \; . \; . \; A(7) = \frac{a(7)}{A(6)}$

*CMFVP* $1.0 \quad B(1) = \frac{b(1)}{1.0} \quad B(2) = \frac{b(2)}{b(1)} \; . \; . \; . \; B(7) = \frac{b(7)}{b(6)}$

From which the estimated log hazard ratio within each period can be calculated

LH − 0.265 − 0.570 . . . − 0.60

$$LH(1) = \ln \frac{\ln(A(1))}{\ln(B(1))} \; . \; . \; . \quad LH(7) = \ln \frac{\ln(A(7))}{\ln(B(7))} \; . \; . \; .$$

The estimated deaths within each period, ignoring censoring, are

| | | | |
|---|---|---|---|
| Deaths | 46.2 | 42.7 . . . | 15 |
| | *D(1) =* | *D(2) =* | *D(7) =* |
| *CAFVP* | *N1*(1-a(1))* | *N1*(a(1)-a(2))* | *N1*(a(6)-a(7))* |
| | + | + | + |
| *CMFVP* | *N2*(1-b(1))* | *N2*(b(1)-b(2)) . . .* | *N2*(b(6)-b(7))* |

where N1 = No of CAFVP patients (107) and N2 = No of CMFVP patients (109).

An estimate of the overall Log Hazard Ratio is then

$$LH = -0.454 = \frac{D(1)*LH(1)}{TD} + \frac{D(2)*LH(2)}{TD} + \; . \; . \; . \quad \frac{D(7)*LH(7)}{TD}$$

where TD is the estimated total deaths = 181

$$TD = D(1) + D(2) + \; . \; . \; D(7)$$

The *P*-value for the comparison of the curves is
$P = 0.01$ (from the published paper)
this corresponds to a 2-sided
standardised normal deviate of 2.58 (*z*)

The standard error of LH (which is always positive) is then approximately

$$\frac{LH}{z} = 0.176 \ (s) \text{ and its variance is } 0.031 \ (s^2)$$

(ii) Combining several log hazard ratios.

Suppose the above trial was the first was the first trial in a series contributing to an overview and let the log hazard ratio calculated above be *LHR(1)* and its variance *V(1)*, a third quantity *W(1)*, the inverse variance weighting factor can be calculated.

If these values for five trials are

| | log Hazard Ratio | Variance | Weight |
|---|---|---|---|
| Trial 1 | − 0.45 | 0.031 | 32.2 |
| Trial 2 | − 0.43 | 0.044 | 22.6 |
| Trial 3 | − 0.85 | 0.311 | 3.2 |
| Trial 4 | 0.06 | 0.133 | 7.5 |
| Trial 5 | − 0.16 | 0.009 | 110.0 |
| *Trial 1* | *LHR(1)* | *V(1)* | *W(1) = 1/V(1)* |
| *Trial 2* | *LHR(2)* | *V(2)* | *W(2) = 1/V(2)* |
| *Trial 3* | . | . | . |
| *Trial 4* | . | . | . |
| *Trial 5* | . | . | . |

Heterogeneity between trials can be tested by calculating

$$G = 4.91$$

$$G = G1 - G2^2/TW$$

which has a Chi-square distribution with 4 (Number of trials-1) degrees of freedom ($P = 0.30$). If this reaches statistical significance then there is evidence that the treatment effect differs between trials, so calculation of a common overall treatment effect is not justified.

The terms in the above equation are given by

G1 = 15.9

$$G1 = W(1)*(LHR(1)^2) + W(2)*(LHR(2)^2) + \; . \; . \; . \; W(5)*(LHR(5)^2)$$

and

G2 = − 43.9

$G2 = W(1)*LHR(1) + W(2)*LHR(2) + \ldots W(5)*LHR(5)$

and where TW is the total of the weights,

TW = 175.5

$TW = W(1) + W(2) + W(3) \ldots + W(5).$

The inverse variance weighted overall log hazard ratio is then

OLHR = − 0.25

$$OLHR = \frac{W(1)*LHR(1)}{TW} + \frac{W(2)*LHR(2)}{TW} + \ldots \frac{W(5)*LHR(5)}{TW}$$

or $OLHR = G2/TW$

This has variance $1/TW$, and its standard error $(SE)$ is given by the square root of $1/TW$. A 95% confidence interval is given by $OLHR \pm (1.96*SE)$.

OLHR = − 0.25 ( − 0.40 - − 0.10)

Exponentiating, this gives

Overall Hazard Ratio = 0.78 (0.67−0.90)

The hypothesis that *OLHR* is zero, i.e. that there is no treatment effect, can be tested by calculating the Chi-square statistic $OLHR^2*TW$ (11.0 in this case) which is compared against a Chi square distribution with one degree of freedom.

(iii) Allowing for censoring.

The assumption made above that there is no censoring will result in greater weight being given to the hazard ratios calculated from the tail of the curve than would occur in practice. Censoring might be allowed for by assuming that a constant proportion *C* are censored in each interval. This will add in an extra term to the calculation of the number of deaths in each interval,

$$D(i) = (1\text{-}C)^i * [N1*(a(i\text{-}1) - a(i)) + N2*(b(i\text{-}1) - b(i))]$$

where i is the interval number.

Assuming that there is 5% censoring in each interval above, the hazard ratio becomes 0.79 (95% CI: 0.68−0.90), there is thus little change.